# Bioinformatics‐based identification of hepatocellular carcinoma‐associated hub genes and assessment of the restorative effect of tannic acid in rat liver exposed to monosodium glutamate

**DOI:** 10.1002/cam4.7404

**Published:** 2024-06-22

**Authors:** Hilal Tosun, Habibe Karadas, Hamid Ceylan

**Affiliations:** ^1^ Department of Molecular Biology and Genetics, Faculty of Science Atatürk University Erzurum Turkey

**Keywords:** bioinformatics, cancer, liver, monosodium glutamate, tannic acid

## Abstract

**Background:**

Hepatocellular carcinoma (HCC) is the most common type of primary liver cancer, occurring mostly in individuals with chronic liver disease, but biomarkers for therapeutic diagnosis and prognosis are lacking. This study aimed to investigate the possible effect of the common food additive monosodium glutamate (MSG) and tannic acid (TA), a phenolic compound, on the key molecular actors responsible for HCC development.

**Methods:**

Eight HCC‐related public microarray datasets (GSE84005, GSE14520, GSE25097, GSE57958, GSE22058, GSE84402, GSE54238, and GSE36376) were extracted from the gene expression omnibus (GEO) database and analyzed to identify differentially expressed genes (DEGs). To make sense of the identified biological data and to identify hub genes, protein–protein interaction (PPI) network and enrichment analysis were performed. The mRNA expression profiles of the identified hub genes, expression changes in different stages of HCC, and their prognostic significances in HCC were determined using GEPIA, UALCAN, and Kaplan–Meier Plotter databases, respectively. Finally, mRNA expression changes of identified hub genes in the liver tissues of rats treated with MSG and TA were measured by the quantitative real‐time PCR (qPCR) method.

**Results:**

Two up‐regulated (*AURKA* and *CCNB2*) and two down‐regulated (*F9* and *CYP2E1*) genes were identified between the HCC tumor and adjacent non‐tumor liver tissue samples. qPCR results showed that the mRNA expression of up‐regulated DEGs involved in HCC development increased significantly in rat liver tissues exposed to MSG, while this increase was remarkably suppressed by TA treatment. It was observed that the mRNA expressions of down‐regulated DEGs involved in HCC development decreased markedly in the presence of MSG, while this decrease was alleviated with TA.

**Conclusion:**

Our results provide new insights into pivotal molecular candidates that should be focused on in future in vivo and in vitro HCC research. Moreover, MSG may play a crucial role in HCC development and progression and TA may be used as a favorable restorative agent in HCC.

## 1 INTRODUCTION

Liver cancer is extremely common and the third leading cause of cancer‐related deaths worldwide. Many risk factors have been associated with hepatocellular carcinoma (HCC), a common form of primary liver cancer in adults.^1^ In addition to non‐alcoholic steatohepatitis (NASH), hepatitis B and C virus (HBV‐HCV) infection, and medical or genetic conditions, personal life habits such as smoking, heavy alcohol use, and nutrition are among the top risk factors.^2^ Nutrition, although an inevitable lifelong environmental factor, is defined as an important modifiable risk factor for HCC.^3^ Numerous epidemiological studies have revealed that dietary nutrients or metabolites have the potential to trigger mechanisms that can lead to cirrhosis, inflammation, and an increased risk of HCC through their potentially toxic effects on the liver.^4^ In addition to predisposing the development of HCC, prominent clinical knowledge has revealed a strong relationship between nutrition and response to HCC treatment.

Human dietary habits have changed greatly in the last 30 years due to the increasing global population and lifestyle changes.^5^ In addition to social developments, multicultural communities that have emerged with the increase in globalization lead to cultural evolution and changes in eating habits.^6^ This situation increased the demand for ready‐made and processed foods, especially in the late 20th century. Nutritional additives are chemical substances that are frequently used in commercial foods today, not only to increase the taste and odor properties of foods but also to increase their shelf life.^7^ One of these, monosodium glutamate (MSG), gives a taste sensation known as umami to processed foods.^8^ Although the Food and Drug Administration (FDA) considers MSG “safe,” many recent studies link MSG to different forms of toxicity.^9^ Moreover, MSG is linked to many health problems such as chronic pulmonary diseases, diabetes, cardiovascular disease, and cancer, which are included in the category of non‐communicable diseases by the World Health Organization (WHO). Numerous in vivo studies have reported that MSG intake below the recommended daily dose for adults (6 g/day)^10^ may lead to various anomalies such as kidney toxicity,^11^ cardiotoxicity,^12^ obesity,^13^ and hepatotoxicity.^14^ We have previously investigated the changes in cortical tissue gene expression promoted by MSG exposure.^15^ Therefore, elucidating the dietary components that negatively affect cellular physiology and the molecular mechanisms of action of these components in pathologies such as HCC may contribute to the development of effective approaches for diagnostic and therapeutic interventions.

Although the effectiveness of chemotherapeutic agents offered to patients with HCC is theoretically strong, long‐term administration leads to clinical paradigms due to side effects such as the development of drug resistance and off‐target toxicity.^16^, ^17^ In this context, in recent years, the use of bioactive natural compounds in the fight against complex diseases such as cancer has come to the fore.^18^ These unusual compounds are naturally occurring compounds that have fewer side effects than designer pharmaceuticals.^19^ Recent reports have revealed that dietary phytochemicals may be low‐cost and less toxic candidates that may prevent the development or progression of disease in different pathologies, including HCC.^20^ Tannic acid (TA) is a natural polyphenol with anticarcinogenic, antimutagenic, anti‐inflammatory, and antioxidant properties. Due to these versatile properties, it has been observed that TA provides promising clinical results against myocardial infarction,^21^ renal failure,^22^, ^23^ and cancer.^24^ TA is also the focus of many studies in terms of its restorative effects on HCC tumor progression.^25^, ^26^

Therefore, this study aimed to identify hepatocellular carcinoma‐associated hub genes using integrated bioinformatics analysis of publicly available GEO microarray datasets. Additionally, this study allows investigation of the effect of MSG exposure as well as TA treatment on the regulation of hepatocellular carcinoma‐associated hub genes in experimental rat models.

## 2 MATERIALS AND METHODS

### 2.1 Collection of transcriptomic data

Publicly available independent liver hepatocellular carcinoma (LIHC)‐associated microarray datasets were downloaded from the Gene Expression Omnibus database (GEO; https://www.ncbi.nlm.nih.gov/geo/ accessed on 12 May 2024).^27^ Eight different expression arrays were selected for this study. The detailed characteristics of the expression profiles are summarized in Table 1.

**TABLE 1:** Characteristics of the datasets used in this study.

GEO accession	Sample size	Platform	Reference
GSE84005	38 HCC tumor samples, 38 adjacent non‐tumor tissues	GPL5175 Affymetrix	[28]
GSE14520	225 HCC tumor samples, 220 adjacent non‐tumor tissues	GPL3921 Affymetrix	[29]
GSE25097	268 HCC tumor samples, 243 adjacent non‐tumor tissues	GPL10687 Affymetrix	[30]
GSE57958	39 HCC tissue samples, 39 adjacent non‐tumor tissue	GPL10558 Illumina	[31]
GSE22058	100 HCC tissue samples, 97 adjacent non‐tumor tissue	GPL6793 Affymetrix	[32]
GSE84402	14 HCC tissue samples, 14 adjacent non‐tumor tissues	GPL570 Affymetrix	[33]
GSE54238	13 HCC tissue samples, 10 adjacent non‐tumor tissues	GPL16955 NimbleGen	[34]
GSE36376	243 HCC tissue samples, 193 adjacent non‐tumor tissues	GPL10558 Illumina	[35]

Abbreviations: GEO, gene expression omnibus; GPL, GEO Platform; HCC, hepatocellular carcinoma.

### 2.2 Differentially expressed genes analysis

Differentially expressed genes (DEGs) between HCC liver tissue and adjacent non‐tumor liver tissue samples were analyzed using GEO2R (http://www.ncbi.nlm.gov/geo2r, accessed on 12 May 2024). *p*‐value < 0.05 and a |log2FC|≥1 were defined as the cut‐off criteria for the upregulated gene identification. |log2FC| ≤ 1 was used as the cut‐off value for downregulated gene identification. Analysis of shared genes in all datasets through the Venn diagram was carried out using The Multiple List Comparator web tool (http://www.molbiotools.com/listcompare.html).

### 2.3 Protein–protein interaction (PPI) network analysis

A protein–protein interaction (PPI) network was created to examine the interaction between genes whose expression changes significantly in tumor tissue compared to adjacent non‐tumor liver tissues and to identify hub genes.^36^, ^37^ The PPI network was constructed using the Search Tool for the Retrieval of Interacting Genes (STRING; https://string‐db.org/, accessed on 12 May 2024) database.^38^ The confidence score ≥0.7 was used to reduce false‐positive interactions. Afterward, the constructed network was visualized and deeply analyzed using Cytoscape software version (3.9.1).^39^ To determine the hub genes, five topological analysis algorithms (Maximal Clique Centrality (MCC), Maximum Neighborhood Component (MNC), Degree, and Betweenness) of the CytoHubba plugin of Cytoscape were used.^40^

### 2.4 Gene ontology and pathway enrichment analysis

Ontological enrichment analysis was performed using the ToppFun module of the ToppGene (https://toppgene.cchmc.org/enrichment.jsp, accessed on 12 May 2024) online bioinformatics resource.^41^ Gene Ontology (GO) analysis, including biological process (BP), molecular function (MF), and, cellular component (CC), and Kyoto Encyclopedia of Genes and Genomes (KEGG) pathway analysis were performed for enrichment analysis of overlapping DEGs.

### 2.5 In silico comparison and validation of candidate hub genes

To compare the mRNA expression differences of hub genes between HCC cancerous liver tissues and corresponding adjacent non‐tumor liver tissues (histologically normal, adjacent to the tumor but beyond the observed aberrations)^42^, ^43^ and to evaluate their prognostic value, the Gene Expression Profiling Interactive Analysis (GEPIA; http://gepia.cancer‐pku.cn/, accessed on 12 May 2024) platform^44^ and the University of ALabama CANcer (UALCAN; https://ualcan.path.uab.edu/, accessed on 12 May 2024) interactive web portal^45^ was used. The correlation between mRNA expression of hub genes and the clinical stage of liver cancer patients, as well as protein expression encoded by hub genes, were also analyzed by UALCAN. Finally, the Kaplan–Meier plotter (https://kmplot.com/analysis/, accessed on 12 May 2024) platform^46^ was utilized to evaluate the prognostic impacts of the hub genes on the overall survival of HCC patients.

### 2.6 Animals and ethics statement

Healthy male Sprague–Dawley rats (*Rattus norvegicus*, male, 180 g ± 10 g, *n* = 24) used in this study were purchased from the Atatürk University Medical Experimental Application and Research Center (Erzurum, Turkiye). Rats were randomly divided into four groups as follows: Control, TA, MSG, and MSG + TA groups. The control group rats were treated with a saline‐only. TA (50 mg/kg)^47^, ^48^ and MSG (2 g/kg)^49^, ^50^ were administered once daily by oral gavage for 21 days. To improve prophylactic effect of the TA, it was administered to the rats in the combined group 1 h before MSG.^51^ All groups were housed in plastic cages under standard conditions (free access to diet and tap water, 22°C ± 3°C air condition, 55% humidity, and 12–12 h lighting). On Day 21, all rats were euthanized under ketamine/xylazine (3:1) anesthesia, and liver tissues were removed immediately and kept at −80°C after washing cold phosphate‐buffered saline. All of the experimental procedures were performed under the guidelines outlined by the National Research Council's Guide for the Care and Use of Laboratory Animals and were approved by the Atatürk University Local Ethics Council for Animal Experiments (Protocol No: 2021‐3/63).

### 2.7 Real‐time PCR analysis

For relative quantification of hub genes mRNA expression, firstly, total RNA was extracted from rat liver using a commercial total RNA extraction kit (Biorad, Hercules, CA, USA) following the manufacturer's instruction. Then, the cDNA library was synthesized using the iScript cDNA synthesis kit (Biorad, Hercules, CA, USA) following the manufacturer's recommendation. To detect hub, liver damage, and apoptotic marker gene expression, pairs of specific primers (Table 2) were designed using the Primer3 (https://bioinfo.ut.ee/primer3‐0.4.0/, accessed on 12 May 2024) online tool.^52^ For relative quantification, SYBR Green‐based qPCR assay was performed using SsoAdvanced™ Universal SYBR® Green Supermix (Biorad, Hercules, CA, USA). *Gapdh* (NM_017008.3) was used as housekeeping control. The comparative ΔΔCt method^53^ was used for the relative quantification of gene expression.

**TABLE 2:** Sequences of primer sets used in quantitative PCR (qPCR).

Gene symbol	Accession ID	Sequence	Tm (°C)
*Aldoa*	M12919.1	F:^5′^‐GGTGGTGTTGTGGGCATTA‐^3′^	60.23
		R:^5′^‐TTGAGTAGTGGTCTCGCCATT‐^3′^	59.75
*Casp3*	NM_12922.2	F: ^5′^‐AGGAGCAGTTTTGTGTGTGTG‐^3′^	59.79
		R: ^5′^‐AGTTTCGGCTTTCCAGTCAG‐^3′^	59.47
*Casp9*	NM_031632.1	F: ^5′^‐TGTGGTGGTGAGCAGAAAGA‐^3′^	60.26
		R: ^5′^‐TCCTGGTATGGGACAGCATC‐^3′^	59.95
*Ccnb2*	NM_001009470.1	F:^5^‐GGGAGGTGGATGTTGAAC‐^3^	59.24
		R:^5^‐ACCTGAGAAGGGTGGTAATG‐^3^	59.55
*Cyp2e1*	NM_031543.2	F:^5^‐TTGTTTCTGCTCCTGTCTGC‐^3^	59.17
		R:^5^‐ATACTGCCAAAGCCAACTGT‐^3^	59.50
*Aurka*	NM_153296.3	F:^5^‐ACCAGATGACAGACCACACG‐^3^	59.58
		R:^5^‐AGACCAAGTGAATGGCTTCG‐^3^	60.25
*F9*	NM_031540.1	F:^5^‐ATCTTCGGCTTTCTCCAGTG‐^3^	59.43
		R:^5^‐CAGTTCGCCATTTACTCTTGC‐^3^	59.90
*Gapdh*	NM_017008.3	F: ^5′^‐AAACCCATCACCATCTTCCA‐^3′^	60.17
		R: ^5′^‐ATACTCAGCACCAGCATCACC‐^3′^	60.16

Abbreviations: F, forward; R, reverse; Tm, melting temperature.

### 2.8 Statistical analysis

Statistical comparison of data obtained from measurements made in triplicate (for each animal and sample) was evaluated with one‐way ANOVA and Tukey's post hoc test using Prism (GraphPad Software, San Diego, CA) software. The statistically significant differences are presented as follows: ^ns^ *p* >0.05 (not significant); *p < 0.05 (significant); **p < 0.01 (very significant); *** or ****p < 0.001 or 0001 (extremely significant).

## 3 RESULTS

### 3.1 Identification of common DEGs of HCC

Up‐regulated and down‐regulated DEGs were screened from GSE84005, GSE14520, GSE25097, GSE57958, GSE22058, GSE84402, GSE54238, and GSE36376, respectively, by GEO2R (Table 3), and intersected genes represented in all eight datasets were identified. A total of 96 common DEGs, including 14 up‐regulated genes and 82 down‐regulated genes, were identified and selected for further analysis (Table S1).

**TABLE 3:** List of differentially expressed genes (DEGs) identified from each dataset.

GEO	Total of DEGs	Up‐regulated DEGs	Down‐regulated DEGs
GSE84005	1007	478	529
GSE14520	1120	515	605
GSE25097	1865	669	1196
GSE57958	416	108	308
GSE22058	1948	697	1251
GSE84402	3512	2186	1326
GSE54238	1888	755	1133
GSE36376	696	429	267

Abbreviation: GEO, gene expression omnibus.

### 3.2 PPI network and cluster analysis

**FIGURE 1:**
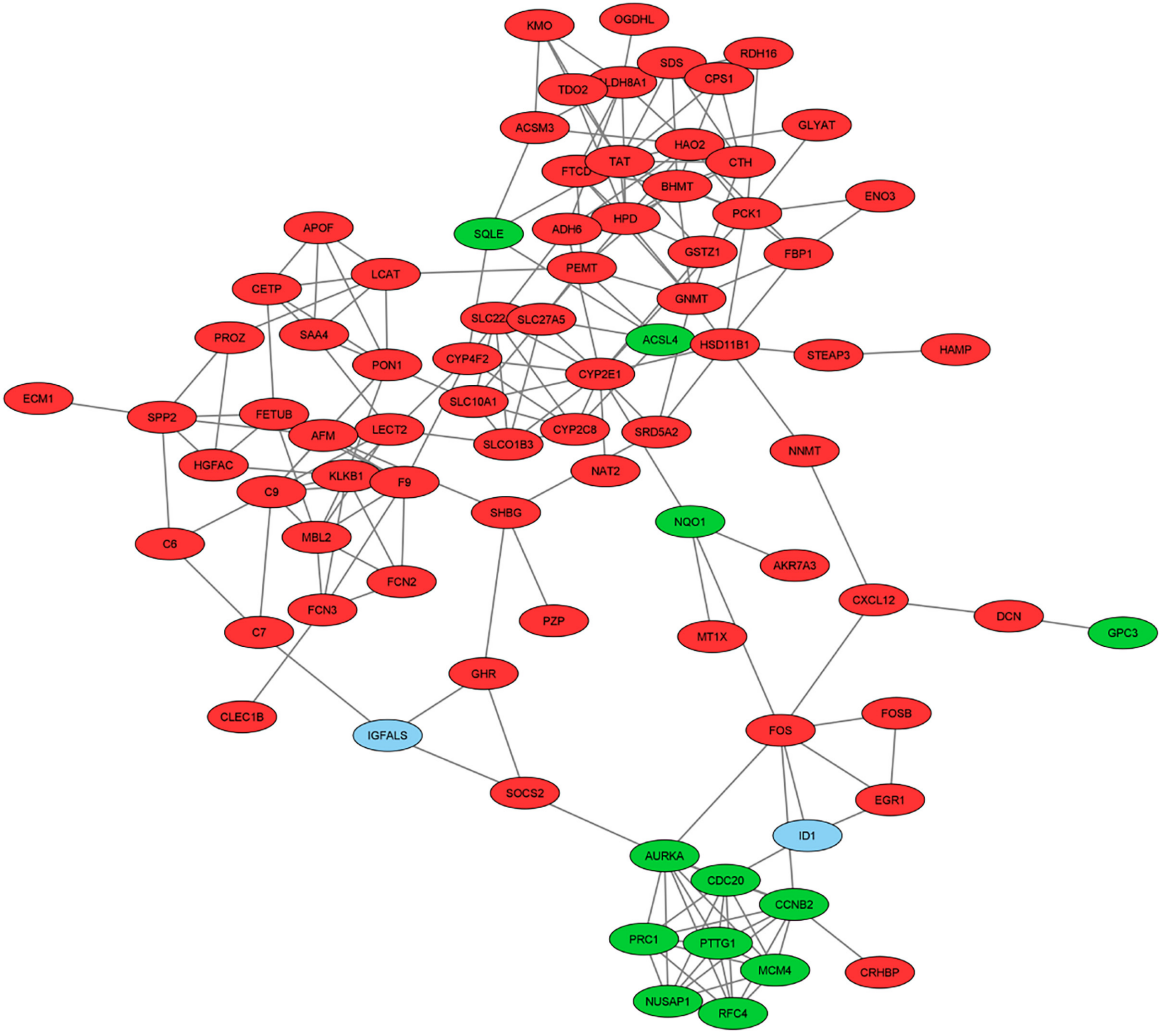
Protein–protein interaction (PPI) network containing all DEGs prominent in HCC and visualized with Cytoscape. Green nodes represent up‐regulated DEGs and red nodes represent down‐regulated DEGs. Blue nodes represent non‐query proteins.

**FIGURE 2:**
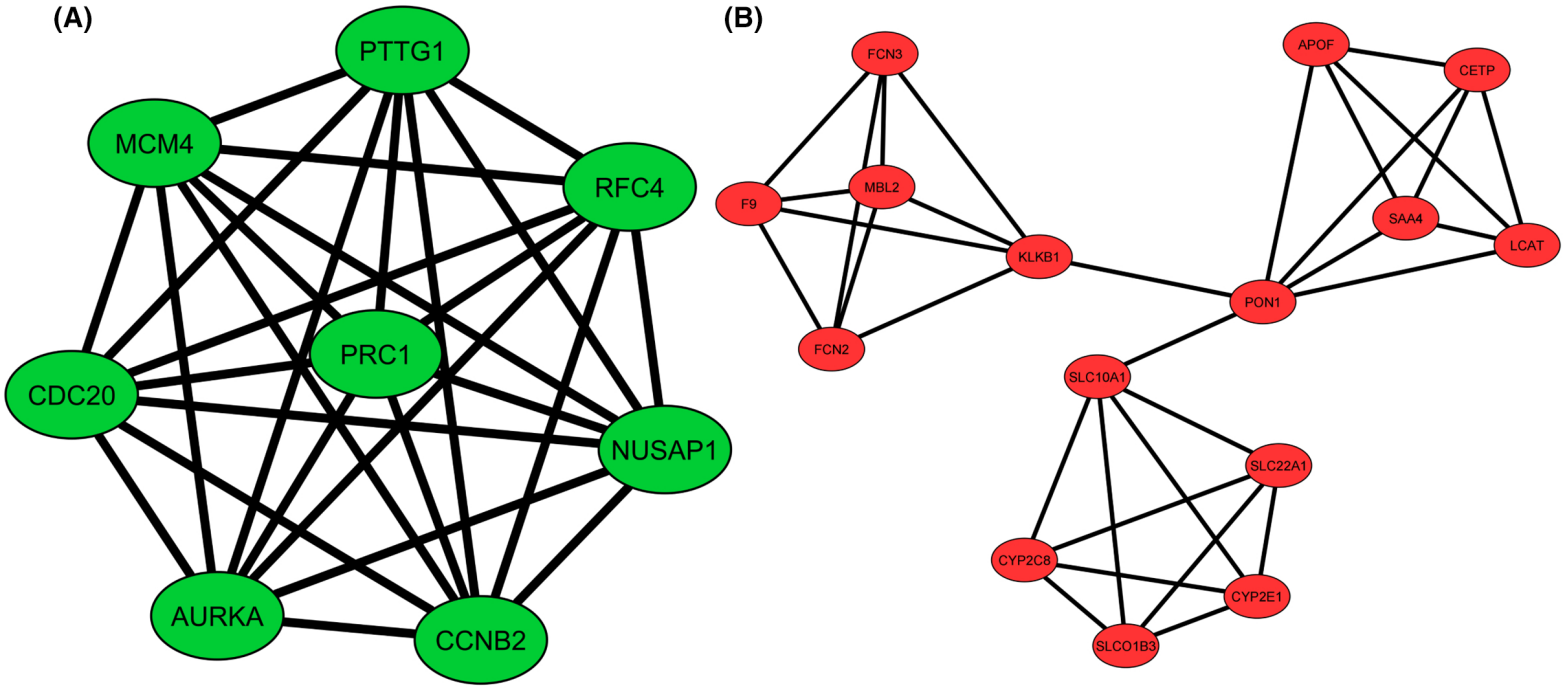
Significant modules identified from the PPI network using the molecular complex detection (MCODE) clustering algorithm. Cluster 1 (A) consisting of up‐regulated DEGs, and Cluster 2 (B) consisting of down‐regulated DEGs. Green nodes represent up‐regulated DEGs and red nodes represent down‐regulated DEGs.

**FIGURE 3:**
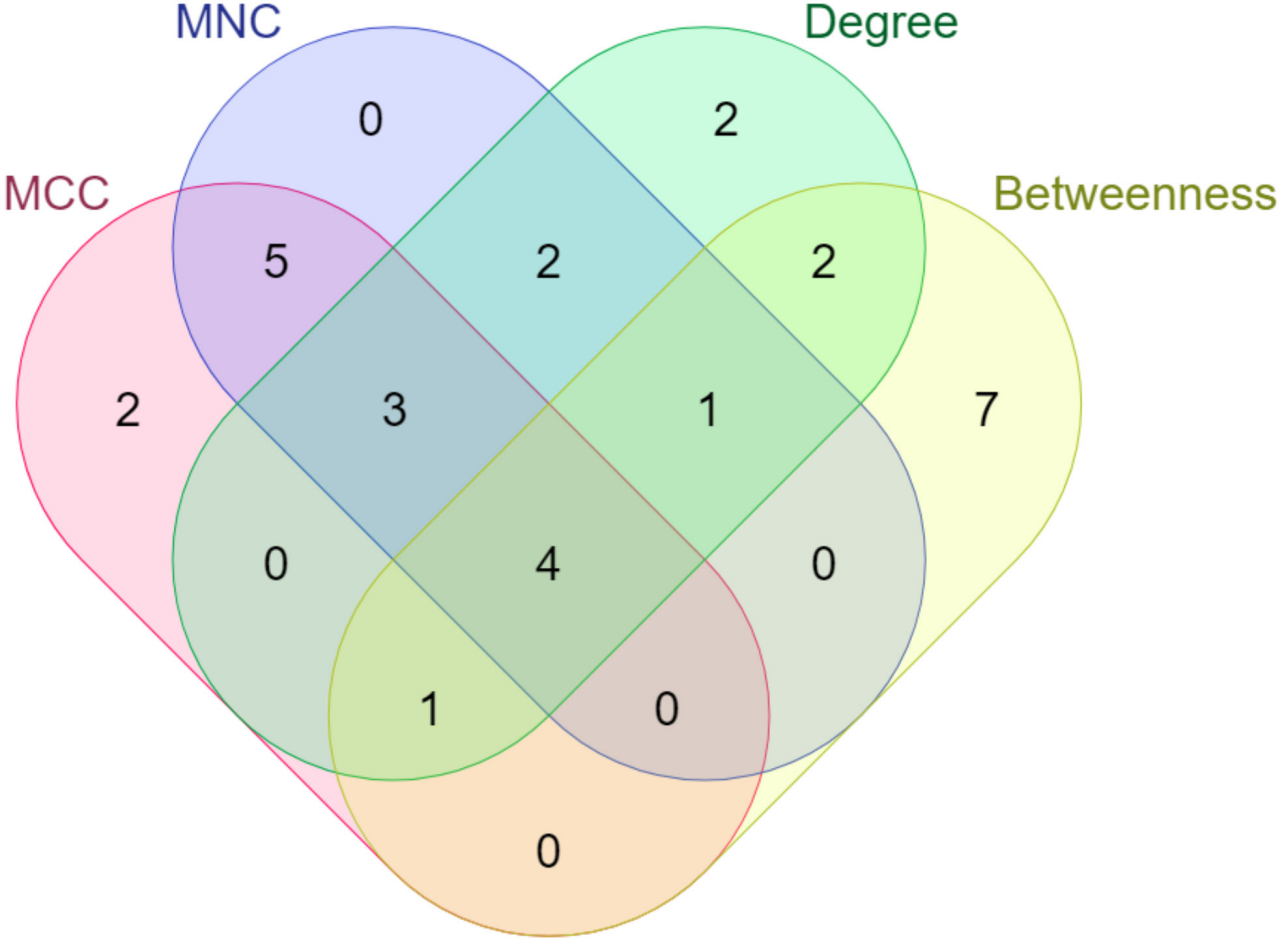
Venn diagram analysis illustrating overlapping DEGs among 4 algorithms of the cytoHubba. Maximal Clique Centrality; MCC, Maximum Neighborhood Component; MNC.

A PPI network was constructed to examine the interaction between genes whose expression was significantly altered in tumor tissue obtained from patients diagnosed with HCC compared to adjacent non‐tumor liver tissue samples. In the PPI network consisting of 82 nodes and 197 interactions (Figure 1), two clusters (Figure 2A,C) were defined based on the degree of importance using the MCODE plug‐in. Then, the top 15 genes that stood out in the five topological algorithms in cytoHubba were selected. Next, hub gene candidates were identified by determining the common genes shared in all algorithms with the Venn diagram (Figure 3). Finally, a total of two up‐regulated genes (*AURKA* and *CCNB2*) and two down‐regulated genes (*F9* and *CYP2E1*) were identified as hub genes.

### 3.3 Gene ontology and pathway enrichment analysis

We conducted gene ontology (GO) and pathway (KEGG) enrichment analysis to further explain potential functions associated with the 14 up‐regulated and 82 down‐regulated DEGs of LIHC. DEGs were classified into three functional categories of GO; molecular functions, biological processes, and cellular components. The pathway analysis results showed that the hub genes were mainly involved in different pathways closely associated with liver cancer progression including retinol metabolism, metabolism of xenobiotics by cytochrome P450, and PPAR signaling pathway (Table S2).

### 3.4 In silico validation of hub genes

**FIGURE 4:**
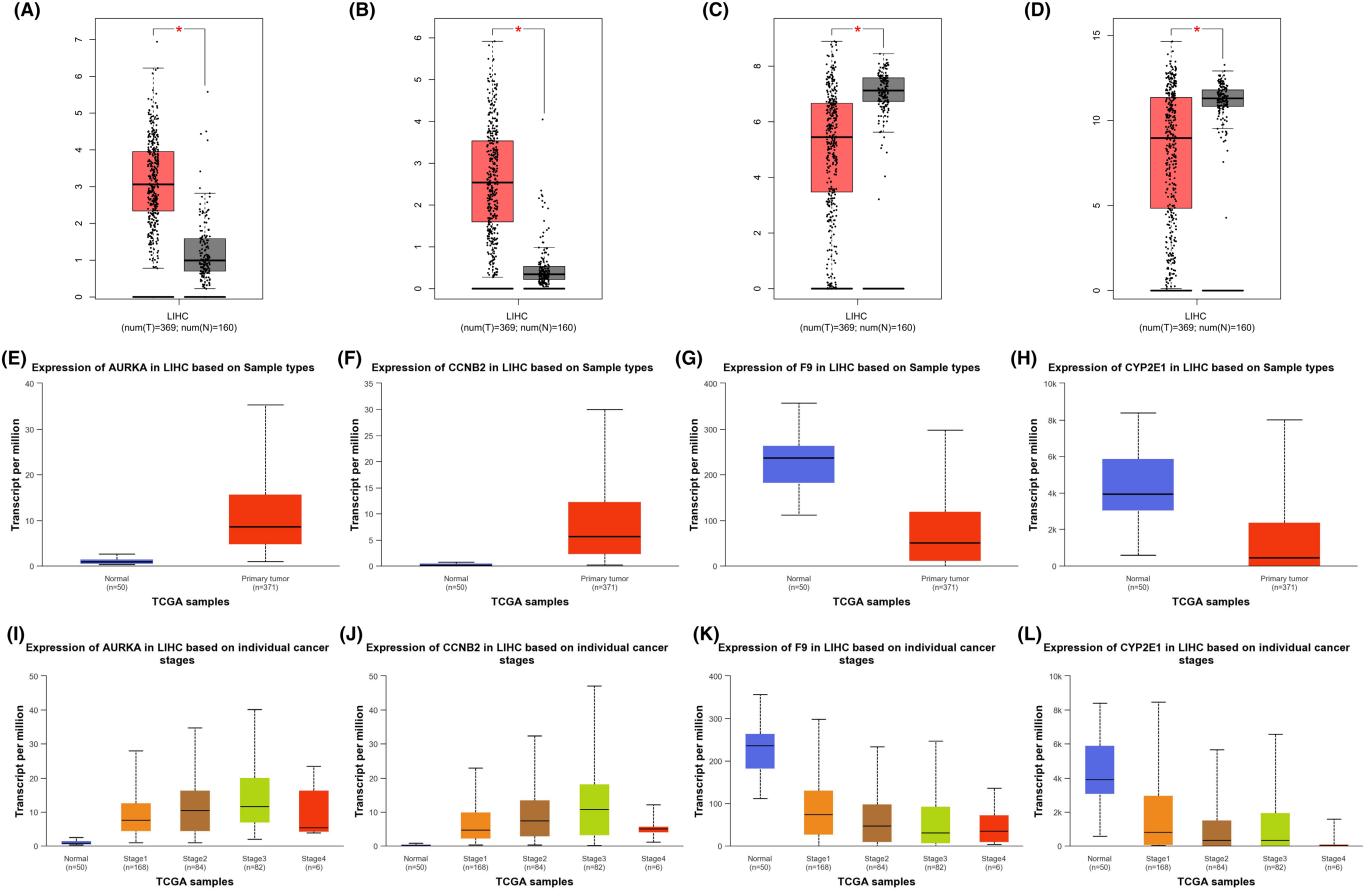
The mRNA expression profiles of hub genes in normal liver tissues and LIHC tissues. The mRNA expression profiles of *AURKA* (A), *CCNB2* (B), *F9* (C), and *CYP2E1* (D) in liver cancer within the GEPIA database. The mRNA expression profiles of *AURKA* (E), *CCNB2* (F), *F9* (G), and *CYP2E1* (H) in liver cancer within the UALCAN database. The mRNA expression profiles of *AURKA* (I), *CCNB2* (J), *F9* (K), and *CYP2E1* (L) in liver cancer patients in different cancer stages. LIHC; liver hepatocellular carcinoma.

Expression differences of the identified hub genes between the liver tissues of patients diagnosed with HCC and adjacent non‐tumor liver tissues were confirmed using GEPIA and UALCAN databases. In parallel with the results of GEO datasets analysis, it was observed that *AURKA* and *CCNB2* mRNA expression up‐regulated in HCC tumor tissues compared to adjacent non‐tumor liver tissues (Figure 4A,B,E, and F). It was confirmed that *F9* and *CYP2E1* mRNA expression was significantly down‐regulated in HCC tumor tissues compared to adjacent non‐tumor liver tissues, again in parallel with the results of GEO datasets analysis (Figure 4C,D,G, and  H). In addition, expression changes of hub genes at different stages of LIHC were also revealed using the UALCAN database. Obtained data showed that *AURKA* and *CCNB2* mRNA expression continued to increase during the advanced stages of LIHC (Figure 4I,J), while *F9* and *CYP2E1* mRNA expression further decreased during the advanced stages of LIHC (Figure 4K,L). Except for CCNB2 (data not available in UALCAN), protein expression levels of AURKA, F9, and CYP2E1 between liver tissues of patients diagnosed with HCC and adjacent non‐tumor liver tissues were observed to parallel mRNA expression levels (Figure S1).

**FIGURE 5:**
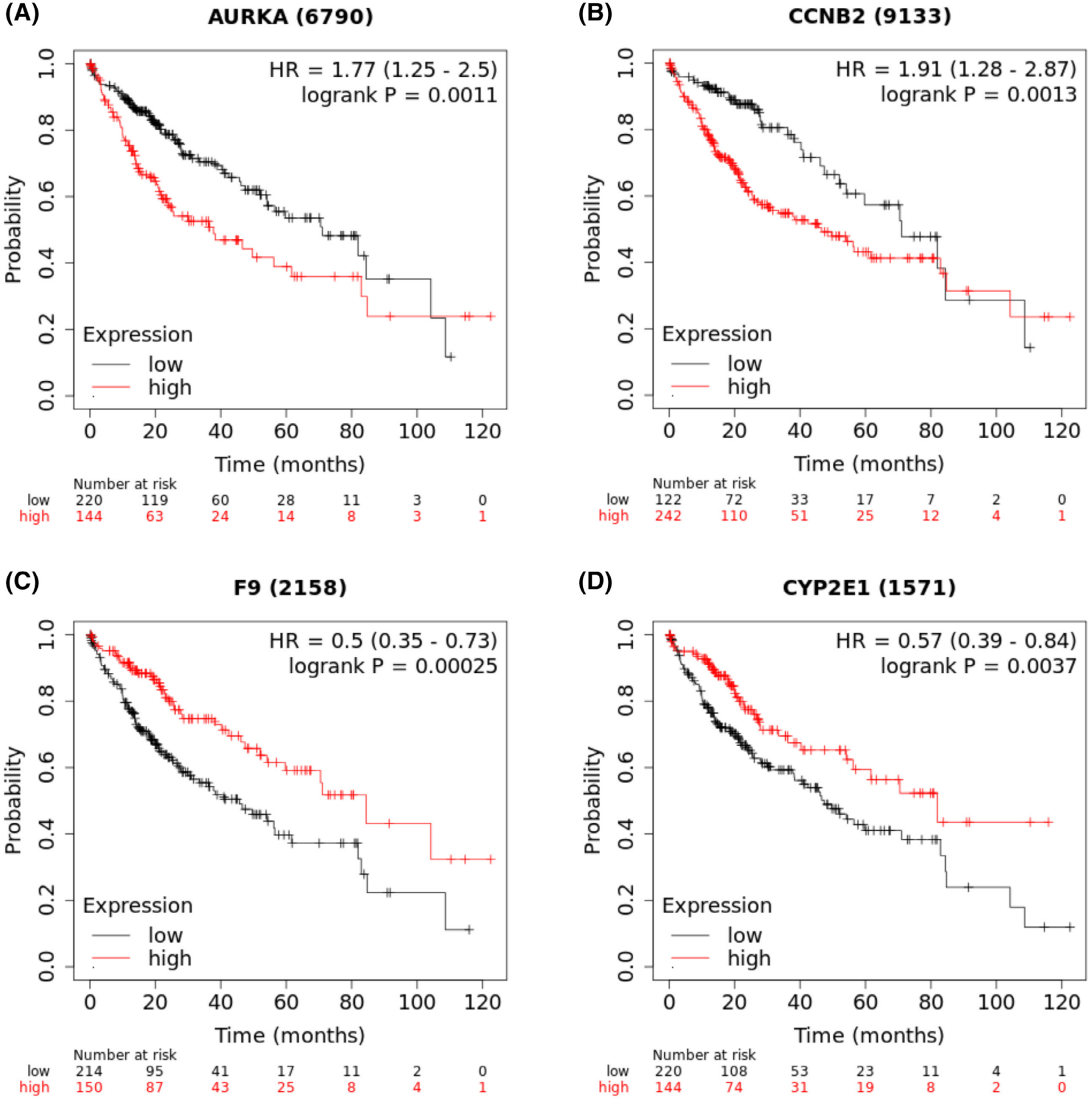
Overall survival (OS) analyses of the hub genes in patients with HCC. (A) *AURKA*, (B) *CCNB2*, (C) *F9*, and (D) *CYP2E1*.

To determine prognostic values of hub genes, OS analysis of LIHC patients was performed using the Kaplan–Meier methodology. The results revealed that higher expression of *AURKA* and *CCNB2* was associated with worse OS rates in LIHC patients (Figure 5A,B). In addition, lower expression of *F9* and *CYP2E1* was also associated with shorter OS in LIHC patients (Figure 5C,D). Detailed information on the prognostic values of the identified hub genes for HCC is listed in Table 4. In summary, these results demonstrated that the identified hub genes may be strong and reliable biomarker candidates for prognosis identification in liver hepatocellular carcinoma patients.

**TABLE 4:** Detailed information on the prognostic values of four hub genes in HCC.

Gene	Modulation in HCC	Probe‐ID	HR	CI	Log‐rank *p‐*value	Median survival low (mo)	Median survival high (mo)
*AURKA*	Up	6790	1.77	1.25–2.5	0.0011	71	37.8
*CCNB2*	Up	9133	1.91	1.28–2.87	0.0013	71	46.6
*F9*	Down	2158	0.5	0.35–0.73	0.00025	45.7	84.4
*CYP2E1*	Down	1571	0.57	0.39–0.84	0.0037	47.4	81.9

Abbreviations: CI, Confidence interval; HCC, hepatocellular carcinoma; HR, Hazard ratios; Mo, month.

### 3.5 Gene expression analysis by RT‐qPCR

**FIGURE 6:**
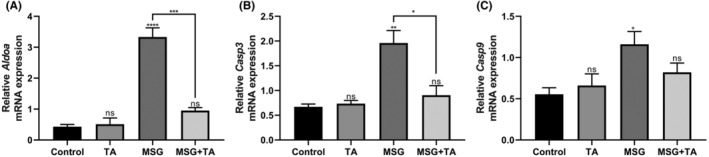
mRNA expression profiles of liver damage and cancer marker genes in the liver tissues of the control vs. treated groups. (A) Relative mRNA expression of *Aldoa*. (B) Relative mRNA expression of *Casp3*. (C) Relative mRNA expression of *Casp9*.

**FIGURE 7:**
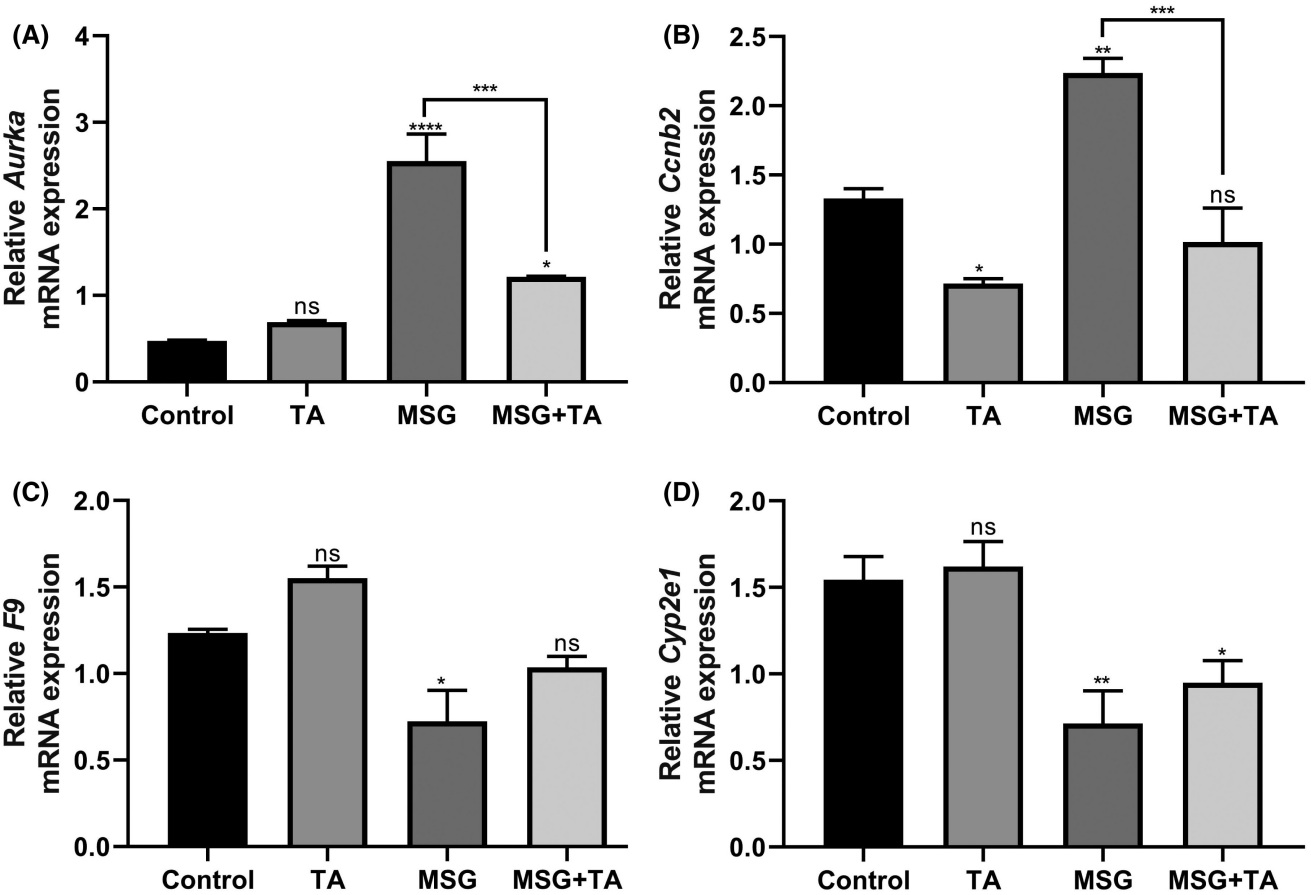
mRNA expression profiles of the hub genes in the liver tissues of the control vs. treated groups. (A) Relative mRNA expression of *Aurka*. (B) Relative mRNA expression of *Ccnb2*. (C) Relative mRNA expression of *F9*. (D) Relative mRNA expression of *Cyp2e1*.

In addition to the mRNA transcript levels of hub genes, we tested whether liver damage occurs after MSG exposure and whether marker genes involved in cancer are triggered. For this purpose, we first examined mRNA expression alterations of *Aldoa* (aldolase, fructose‐bisphosphate A)^54^ to test whether MSG‐induced liver damage occurred. There was a significant increase in *Aldoa* expression in rat liver tissues treated with MSG alone compared to the control group, however, this increase was remarkably inhibited with the help of TA (Figure 6A). Next, we examined whether MSG triggers cell death in the liver tissue and the protection potential of TA against MSG by analyzing caspase 3 and 9 gene expression. As shown in Figure 6B,C, MSG exposure strongly boosted the *Casp3* and *Casp9* expression almost 4 and 3‐fold, respectively. Meanwhile, it was observed that the co‐administration of TA attenuated this rigid increase. Finally, the effects of MSG and TA, alone or in combination with each other, on the mRNA transcript levels of hub genes were examined. As shown in Figure 7A,B, it was observed that the relative mRNA expressions of *Aurka* and *Ccnb2*, which are up‐regulated in LIHC, increased significantly with MSG stimulation, however, this increase efficiently reverted by TA treatment. The relative mRNA expressions of *F9* and *Cyp2e1*, which are down‐regulated in LIHC, were also suppressed by MSG exposure and re‐induced by simultaneous TA treatment (Figure 7C,D).

## 4 DISCUSSION

Liver cancer (also known as hepatocellular carcinoma; HCC) is a common type of cancer accompanied by high mortality and poor prognosis. Due to its high prevalence, diagnosis, and treatment studies on HCC attract the attention of researchers. Therefore, the identification of critical biomarkers underlying complex pathology is considered important in preventing recurrence and metastasis. Identification of differential genes in non‐communicable diseases such as cancer caused by cumulative dysregulation in gene expression is now possible with integrated bioinformatics analysis approaches. In this study, not only candidate hub genes involved in HCC were identified by analysis of publicly available GEO datasets but also the diet‐related alteration of these genes was investigated in experimental rat models. In the present study, the eight liver cancer‐related datasets (GSE84005, GSE14520, GSE25097, GSE57958, GSE22058, GSE84402, GSE54238, and GSE36376) extracted from GEO, and a total of 96 DEGs (14 up‐regulated and 82 down‐regulated) between HCC and adjacent non‐tumor liver tissue samples were screened. Moreover, to narrow down the number of target genes and identify HHC‐related critical central genes, the PPI network was created and the most important proteins directly connected were determined. Consequently, 2 up‐regulated (*AURKA* and *CCNB2*) and 2 down‐regulated (*F9* and *CYP2E1*) DEGs were identified as hub genes, and their expression changes in tumor tissues and the impact of these changes on HCC prognosis were confirmed by in silico tools.

The increasing trend of ready‐made and packaged food consumption on a global scale makes exposure to food additives used to increase the shelf life and taste of such processed food products inevitable.^55^ Monosodium glutamate (MSG), used for this purpose, can affect the risk of common health problems such as heart diseases, type 2 diabetes, and cancer, especially by contributing to metabolic changes, which are considered one of the hallmarks of cancer.^56^, ^57^ Improper metabolic reprogramming in critical processes in the liver, such as energy (adenosine triphosphate; ATP) production, glucose metabolism, and amino acid and fatty acid metabolism supports the ability for tumorigenesis, proliferation, and metastasis in HCC.^58^, ^59^ Therefore, it is considered very important to determine general dietary patterns by considering these risks. It has been demonstrated that MSG administration (at a dose consistent with the human ADI, acceptable daily intake) led to fertility impairment, increased weight gain, and remarkable changes in major organs such as mild portal inflammation in the liver and periglomerular fibrosis and interstitial nephritis in the kidney of Wistar rats.^60^ The study by Wang et al.^61^ documented that MSG can lead to reproductive toxicity in male mice. Meanwhile, similar reports also showed that MSG disrupted the antioxidant system in testicular tissue, induced apoptosis, and caused degeneration in spermatogenesis.^62^, ^63^ Other studies also postulated that MSG can cause derangement in cardiac functional indicators,^64^ and can cause chemical brain damage and cognitive impairments.^65^, ^66^ Although international regulatory agencies (US Food and Drug Administration; FDA, the European Food Safety Association; EFSA, and Joint FAO/WHO Expert Committee on Food Additives; JECFA) evaluate MSG as a GRAS (generally recognized as safe) substance with established limits,^67^, ^68^ mentioned preclinical studies emphasize that MSG exposure, especially with chronic consumption, causes many different organ toxicities and therefore poses a real health risk.^8^, ^69^ Some authors argue that this paradigm has emerged because GRAS determination criteria and methodologies, especially for experience‐based procedures, have not been updated in accordance with today's toxicity determination tests and technologies.^70^, ^71^, ^72^

To date, a large number of independent studies have conducted numerous studies on Tannic acid (TA), a plant‐derived polyphenol, and shown that TA has pharmacological effects such as antioxidant, antimicrobial, anti‐inflammatory, anticarcinogenic, and antimutagenic.^73^ In addition to its multifunctional biological potential, TA has attracted great attention because it is cheap, easily accessible, and can be taken in the daily diet.^74^ Although not many studies evaluate MSG and TA together, reports have revealed that TA can effectively protect against MSG‐induced toxic outcomes. Calis et al.^75^ observed that TA pretreatment reduced lipid peroxidation and blood glucose, which are increased by MSG exposure, and also significantly increased blood superoxide dismutase (SOD) levels. Similar studies demonstrated that serum levels of alanine aminotransferase (ALT) and aspartate aminotransferase (AST), crucial indicators for diagnosing and assessing liver diseases, and apoptotic marker expression were significantly boosted in MSG‐treated rats, however, hepatic toxicity induced by MSG was ameliorated by TA.^76^, ^77^ Consistent with previous studies, our results show that MSG exposure has a negative effect on liver damage (*Aldoa*) and apoptotic (*Casp3* and *Casp9*) marker gene expression (Figure 6). ALDOA (aldolase A), a key enzyme of glycolysis metabolism, is one of the most abundant glycolytic enzymes in tumor cells and has been shown to play an important role in promoting tumor growth and metastasis in many different types of cancer.^78^ Recent studies also show that high *Aldoa* expression plays a role in the regulation of cancerization processes (including HCC cell proliferation and migration) and the cell death signaling network (together with *Casp3* and *Casp9*), and it is proposed that it may lead to poor prognosis due to its effect on immune infiltration.^79^, ^80^ Our results suggest that MSG adversely affects these mechanisms that contribute to HCC, but TA treatment can potentially stimulate these underlying actors.

The cell cycle is a tightly controlled mechanism as it is the basis of biological activities that affect the cell's fate, such as correct duplication of genomic DNA, initiation of division at the appropriate point, and genetic diversity.^81^ Defects in the cell cycle interrupt critical signal transduction and lead to abnormal cell division, which is an important hallmark of cancer.^82^ Therefore, the aberrant regulation of cell cycle regulation and metastasis‐related genes remarkably affects tumor prognosis. Aurora kinase A (AURKA), a member of the fundamental aurora kinase family, plays a role in chromosome segregation and cell cycle management by regulating spindle formation during cell division.^83^ AURKA and its downstream target genes regulate the HCC development and progression through various pathways including the FOXO signaling pathway, PI3K/AKT pathway, TP53 pathway, and NFKB pathway.^84^, ^85^ Numerous studies have highlighted dysregulation of *AURKA* expression in different types of cancer, such as breast,^86^ ovarian,^87^ prostate,^88^ and liver cancer.^89^ Previous studies have shown that *AURKA* is overexpressed in HCC and this expression profile is associated with poor prognosis.^90^ Chen et al.^91^ reported that *AURKA* was abnormally expressed and involved in HCC metastasis. Jeng et al.^92^ revealed that *Aurora A* overexpression in HCC was associated with cancer stage and grade, thus management of its expression is a promising therapeutic target for HCC. Cyclin B2 (CCNB2), a member of the B‐type cyclin family, is another tight regulator of the G2/M checkpoint, which is responsible for DNA repair and genome integrity.^93^ Therefore, the unusual activity of cyclin family proteins such as CCNB2 leads to mutations in genes, structural changes in the chromosome, and abnormal cell proliferation, ultimately leading to tumor development.^94^ Previous studies have reported a correlation between *CCNB2* overexpression and the development and progression of different malignancies. Li et al.^95^ suggested that CCNB2 expression, which is higher in HCC than in normal liver tissues, is associated with cell proliferation and migration, therefore CCNB2 is a prognostic factor for HCC. Another study of the same group revealed that the tumor development rate was reduced and apoptosis was induced in *CCNB2*‐siRNA lentivirus infected Huh‐7 hepatoma cells transfected mice compared to the control group, therefore *CCNB2* silencing could prevent HCC formation.^96^

Coagulation Factor IX (F9) is an important plasma protein that participates in the blood clotting pathway by being synthesized in the liver and secreted into the plasma.^97^ Carpintero et al.^98^ confirmed that coagulation factor IX is a key regulator of senescence, which halts cancer development by preventing the malignant transformation of healthy cells and its mRNA is up‐regulated during senescence. They identified that the downregulation of F9 inhibits senescence induction, and demonstrated that this blocking reverted by recombinant F9 treatment. Ishikawa et al.^99^ found that coagulation factor IX is involved in the induction of apoptosis and cell adhesion attenuation. Plasma analyses of patients diagnosed with stage III HCC also showed that factor IX activity levels were significantly reduced compared to healthy controls.^100^ These findings indicate that suppression of F9 in senescence may lead to uncontrolled growth of cancer cells and may be a proliferative advantage for tumor cells.

Cytochrome P450 (CYP) enzymes are a hemoprotein superfamily involved in the metabolism of xenobiotics such as procarcinogens. Due to these properties, CYPs play a role in the occurrence and progression of different pathologies such as cardiovascular diseases, diabetes, neurodegenerative diseases, and cancer.^101^ Previous studies found that low expression of CYP2E1 contributes to HCC susceptibility. Fan et al.^102^ revealed that CYP2E1 is down‐regulated in tumor tissues compared with adjacent non‐tumor tissues and was associated with the malignant tumor phenotype. Another study showed that 15 CYP family member genes, including *CYP2E1*, were significantly suppressed in HCC compared to healthy tissues.^103^ It has also been reported that hepatoma cell growth is promoted by suppressing CYP2E1 gene expression through HNF4α (Hepatocyte Nuclear Factor 4α), which is an important regulator of hepatic differentiation.^104^ In this perspective, we speculate that MSG may play an important role in the development and progression of HCC by affecting the proper regulation of identified hub genes, in addition, TA may accurately stimulate such a mechanism underlying HCC development. To the best of our knowledge, this is the first study to consider monosodium glutamate and tannic acid together in the hepatocellular carcinoma axis.

## 5 CONCLUSION

In conclusion, this study indicates that hub genes and pathways found through a comprehensive integrated bioinformatics analysis may be associated with HCC development and may be prospective biomarkers and restorative targets to focus on in the next studies. Furthermore, the mRNA alteration of these six HCC‐associated key genes was confirmed in the liver tissue of rats exposed to MSG and also treated with TA. The findings of our research need further in vivo experimental approval but can be declared to be helpful arguments for alleviating HCC development and progression.

## AUTHOR CONTRIBUTIONS

**Hilal Tosun:** Data curation (equal); formal analysis (equal); methodology (equal). **Habibe Karadas:** Data curation (equal); formal analysis (equal); methodology (equal). **Hamid Ceylan:** Conceptualization (lead); data curation (equal); formal analysis (equal); writing – original draft (lead); writing – review and editing (lead).

## FUNDING INFORMATION

This research did not receive any specific grant from funding agencies in the public, commercial, or not‐for‐profit sectors.

## CONFLICT OF INTEREST STATEMENT

There are no financial conflicts of interest to disclose.

## ETHICS STATEMENT

All of the experimental procedures were performed under the guidelines outlined by the National Research Council's Guide for the Care and Use of Laboratory Animals and were approved by the Atatürk University Local Ethics Council for Animal Experiments (Protocol No: 2021‐3/63).

## Supporting information


Data S1.

## Data Availability

All relevant data analyzed during the current study are available in the GEO repository.
