# Supplementary material for: Bioinformatics‐based identification of hepatocellular carcinoma‐associated hub genes and assessment of the restorative effect of tannic acid in rat liver exposed to monosodium glutamate
Source: Cancer Med. 2024 Jun 22;13(12):e7404. doi: 10.1002/cam4.7404 (PMC11192999; doi:10.1002/cam4.7404)
Supplement: Supplementary file 1 — Data S1. [file CAM4-13-e7404-s001.docx]

**Supplementary File 1**

**Table S1.** Common up-regulated and down-regulated DEGs in GEOs derived from HCC tumor tissues and adjacent non-tumor tissues. **HCC**; hepatocellular carcinoma, **DEGs**; differentially expressed genes, **GEO**; gene expression omnibus.

| Shared up-regulated (*p*-value <0.05, \|log2FC\| ≥ 1) genes in all GEOs | | | | |
| --- | --- | --- | --- | --- |
| *AURKA* | *SQLE* | *PTTG1* | *CCNB2* | *PRC1* |
| *MCM4* | *GPC3* | *MUC13* | *ACSL4* | *SPINK1* |
| *CDC20* | *RFC4* | *NQO1* | *NUSAP1* |  |
| Shared down-regulated (*p*-value <0.05, \|log2FC\| ≤ 1) genes in all GEOs | | | | |
| *BBOX1* | *FAM134B* | *ACSM3* | *GHR* | *CLEC1B* |
| *BHMT* | *SHBG* | *ID1* | *CRHBP* | *SRD5A2* |
| *AKR7A3* | *KLKB1* | *FETUB* | *CYP2C9* | *NNMT* |
| *APOF* | *SDS* | *SLC10A1* | *CYP4F2* | *NAT2* |
| *FBP1* | *CETP* | *ENO3* | *SPP2* | *HAO2* |
| *SAA4* | *GSTZ1* | *CPS1* | *FXYD1* | *KMO* |
| *FOSB* | *MARCO* | *LCAT* | *HAMP* | *DCN* |
| *HGFAC* | *PZP* | *C9* | *PROZ* | *MBL2* |
| *SLCO1B3* | *PON1* | *STEAP3* | *DNASE1L3* | *F9* |
| *IGFALS* | *EGR1* | *ECM1* | *RDH5* | *FCN2* |
| *SLC22A1* | *PCK1* | *CYP2E1* | *CTH* | *RND3* |
| *RDH16* | *AFM* | *DNAJC12* | *MT1X* | *C7* |
| *HPD* | *TAT* | *FOS* | *ALDH8A1* | *CXCL12* |
| *GNMT* | *GLYAT* | *LECT2* | *SOCS2* | *CYP2C8* |
| *PEMT* | *FCN3* | *GBA3* | *ADH6* | *TDO2* |
| *HSD11B1* | *VIPR1* | *FTCD* | *OGDHL* | *CYP3A4* |
| *SLC27A5* | *C6* |  |  |  |

**Table S2.** GO (Gene Ontology) and KEGG (Kyoto Encyclopedia of Genes and Genomes) pathway enrichment analysis results of genes found to be differentially expressed (*p*-value <0.05, |log2FC| ≥ 1 and |log2FC| ≤ 1) between HCC tumor tissues compared with adjacent non-tumor tissues. The top 20 significant GO and pathway terms were listed. **HCC**; hepatocellular carcinoma, **MF**; Molecular Function, **BP**; Biological Process, and **CC**; Cellular Component.

|  | Term ID | Term description | *p-*value | *q*-value FDR B&H | Hit in Query List |
| --- | --- | --- | --- | --- | --- |
| MF: Molecular Function | | | | | |
|  | GO:0016491 | oxidoreductase activity | 2.205E-12 | 1.261E-9 | *ADH6, GSTZ1, CYP2C8, CYP2C9, RDH16, CYP2E1, CYP3A4, HPD, SQLE, SRD5A2, NQO1, AKR7A3, ALDH8A1, RDH5, STEAP3, OGDHL, CYP4F2, TDO2, HSD11B1, BBOX1, HAO2, KMO, SLC27A5* |
|  | GO:0016614 | oxidoreductase activity, acting on CH-OH group of donors | 4.055E-7 | 7.519E-5 | *ADH6, RDH16, SRD5A2, AKR7A3, RDH5, CYP4F2, HSD11B1, HAO2* |
|  | GO:0070330 | aromatase activity | 5.258E-7 | 7.519E-5 | *CYP2C8, CYP2C9, CYP2E1, CYP3A4, CYP4F2* |
|  | GO:0004497 | monooxygenase activity | 2.002E-6 | 2.290E-4 | *CYP2C8, CYP2C9, CYP2E1, CYP3A4, SQLE, CYP4F2, KMO* |
|  | GO:0008392 | arachidonic acid epoxygenase activity | 4.603E-6 | 3.208E-4 | *CYP2C8, CYP2C9, CYP2E1, CYP4F2* |
|  | GO:0033695 | oxidoreductase activity, acting on CH or CH2 groups | 5.609E-6 | 3.208E-4 | *CYP2C8, CYP2C9, CYP3A4* |
|  | GO:0034875 | caffeine oxidase activity | 5.609E-6 | 3.208E-4 | *CYP2C8, CYP2C9, CYP3A4* |
|  | GO:0008391 | arachidonic acid monooxygenase activity | 8.742E-6 | 4.546E-4 | *CYP2C8, CYP2C9, CYP2E1, CYP4F2* |
|  | GO:0019842 | vitamin binding | 1.269E-5 | 6.049E-4 | *AFM, OGDHL, CTH, FTCD, GNMT, SDS, TAT* |
|  | GO:0101020 | estrogen 16-alpha-hydroxylase activity | 1.636E-5 | 7.061E-4 | *CYP2C8, CYP2C9, CYP3A4* |
|  | GO:0005506 | iron ion binding | 1.728E-5 | 7.061E-4 | *CYP2C8, CYP2C9, CYP2E1, CYP3A4, HPD, CYP4F2, BBOX1* |
|  | GO:0033764 | steroid dehydrogenase activity, acting on the CH-OH group of donors, NAD or NADP as acceptor | 1.938E-5 | 7.389E-4 | *RDH16, SRD5A2, RDH5, HSD11B1* |
|  | GO:0106429 | 11-cis-retinol dehydrogenase | 2.205E-5 | 7.883E-4 | *RDH16, RDH5* |
|  | GO:0030414 | peptidase inhibitor activity | 2.960E-5 | 9.960E-4 | *PTTG1, GPC3, SPİNK1, SPP2, SLCO1B3, PZP, FETUB* |
|  | GO:0008401 | retinoic acid 4-hydroxylase activity | 3.571E-5 | 1.125E-3 | *CYP2C8, CYP2C9, CYP3A4* |
|  | GO:0016229 | steroid dehydrogenase activity | 3.737E-5 | 1.125E-3 | *RDH16, SRD5A2, RDH5, HSD11B1* |
|  | GO:0016616 | oxidoreductase activity, acting on the CH-OH group of donors, NAD or NADP as acceptor | 4.161E-5 | 1.190E-3 | *ADH6, RDH16, SRD5A2, AKR7A3, RDH5, HSD11B1* |
|  | GO:0005496 | steroid binding | 4.521E-5 | 1.231E-3 | *CYP3A4, AFM, CETP, SHBG, APOF, HSD11B1* |
|  | GO:0031406 | carboxylic acid binding | 6.282E-5 | 1.633E-3 | *FCN2, CYP4F2, TDO2, FTCD, CPS1, GNMT, PCK1, TAT* |
|  | GO:0098603 | selenol Se-methyltransferase activity | 6.595E-5 | 1.640E-3 | *GNMT, NNMT* |
| BP: Biological Process | | | | | |
|  | GO:0006082 | organic acid metabolic process | 1.312E-17 | 3.487E-14 | *GHR, ADH6, ACSL4, GLYAT, GSTZ1, CYP2C8, CYP2C9, ACSM3, FBP1, RDH16, PEMT, CYP2E1, CYP3A4, HPD, SRD5A2, ALDH8A1, PON1, OGDHL, CYP4F2, CTH, TDO2, FTCD, CPS1, GNMT, ENO3, HAO2, SDS, PCK1, TAT, KMO, SLC27A5, BHMT* |
|  | GO:0019752 | carboxylic acid metabolic process | 5.529E-17 | 7.347E-14 | *ADH6, ACSL4, GLYAT, GSTZ1, CYP2C8, CYP2C9, ACSM3, FBP1, RDH16, PEMT, CYP2E1, CYP3A4, HPD, SRD5A2, ALDH8A1, PON1, OGDHL, CYP4F2, CTH, TDO2, FTCD, CPS1, GNMT, ENO3, HAO2, SDS, PCK1, TAT, KMO, SLC27A5, BHMT* |
|  | GO:0043436 | oxoacid metabolic process | 1.019E-16 | 9.032E-14 | *ADH6, ACSL4, GLYAT, GSTZ1, CYP2C8, CYP2C9, ACSM3, FBP1, RDH16, PEMT, CYP2E1, CYP3A4, HPD, SRD5A2, ALDH8A1, PON1, OGDHL, CYP4F2, CTH, TDO2, FTCD, CPS1, GNMT, ENO3, HAO2, SDS, PCK1, TAT, KMO, SLC27A5, BHMT* |
|  | GO:0009410 | response to xenobiotic stimulus | 1.074E-12 | 7.140E-10 | *ADH6, GLYAT, NAT2, CYP2C8, CYP2C9, SLC10A1, FBP1, PEMT, CYP2E1, CYP3A4, FOS, FOSB, SLC22A1, SRD5A2, NQO1, SLCO1B3, CYP4F2, CPS1, NNMT, CRHBP* |
|  | GO:0044283 | small molecule biosynthetic process | 4.986E-12 | 2.335E-9 | *CYP2C8, CYP2C9, ACSM3, FBP1, RDH16, CYP2E1, EGR1, CYP3A4, SRD5A2, ALDH8A1, RDH5, CTH, CPS1, GNMT, NNMT, ENO3, SDS, PCK1, KMO, SLC27A5, BHMT* |
|  | GO:1901605 | alpha-amino acid metabolic process | 5.271E-12 | 2.335E-9 | *GLYAT, GSTZ1, PEMT, HPD, ALDH8A1, CTH, TDO2, FTCD, CPS1, GNMT, SDS, TAT, KMO, BHMT* |
|  | GO:0071466 | cellular response to xenobiotic stimulus | 1.319E-11 | 5.009E-9 | *ADH6, GLYAT, NAT2, CYP2C8, CYP2C9, SLC10A1, FBP1, CYP2E1, CYP3A4, SLC22A1, NQO1, SLCO1B3, CYP4F2, CRHBP* |
|  | GO:0006629 | lipid metabolic process | 6.627E-11 | 2.202E-8 | *ADH6, SOCS2, GBA3, ACSL4, CYP2C8, CYP2C9, ACSM3, RDH16, PEMT, CYP2E1, EGR1, CYP3A4, CETP, SQLE, SRD5A2, APOF, AKR7A3, ALDH8A1, PON1, RDH5, CYP4F2, CTH, HSD11B1, LCAT, CPS1, HAO2, SDS, PCK1, SLC27A5* |
|  | GO:0032787 | monocarboxylic acid metabolic process | 1.234E-10 | 3.611E-8 | *ADH6, ACSL4, GLYAT, CYP2C8, CYP2C9, ACSM3, FBP1, RDH16, CYP2E1, CYP3A4, ALDH8A1, OGDHL, CYP4F2, FTCD, ENO3, HAO2, SDS, PCK1, KMO, SLC27A5* |
|  | GO:1901615 | organic hydroxy compound metabolic process | 1.359E-10 | 3.611E-8 | *ADH6, CYP2C8, CYP2C9, RDH16, CYP2E1, CYP3A4, CETP, SQLE, SRD5A2, APOF, NQO1, AKR7A3, PON1, RDH5, CYP4F2, LCAT, HAO2, PCK1, SLC27A5* |
|  | GO:0008202 | steroid metabolic process | 2.575E-10 | 6.221E-8 | *CYP2C8, CYP2C9, RDH16, CYP2E1, EGR1, CYP3A4, CETP, SQLE, SRD5A2, APOF, PON1, RDH5, HSD11B1, LCAT, SLC27A5* |
|  | GO:0006520 | amino acid metabolic process | 3.170E-10 | 7.022E-8 | *GLYAT, GSTZ1, PEMT, HPD, ALDH8A1, CTH, TDO2, FTCD, CPS1, GNMT, SDS, TAT, KMO, BHMT* |
|  | GO:0120254 | olefinic compound metabolic process | 8.496E-10 | 1.737E-7 | *ADH6, CYP2C8, CYP2C9, RDH16, CYP2E1, EGR1, CYP3A4, SRD5A2, ALDH8A1, RDH5, CYP4F2* |
|  | GO:0044282 | small molecule catabolic process | 1.251E-9 | 2.375E-7 | *ADH6, GSTZ1, CYP3A4, HPD, ALDH8A1, PON1, CYP4F2, TDO2, FTCD, ENO3, HAO2, SDS, PCK1, TAT, KMO* |
|  | GO:0006805 | xenobiotic metabolic process | 1.385E-9 | 2.454E-7 | *GLYAT, NAT2, CYP2C8, CYP2C9, CYP2E1, CYP3A4, SLC22A1, NQO1, SLCO1B3, CYP4F2* |
|  | GO:0016054 | organic acid catabolic process | 6.390E-9 | 9.945E-7 | *GSTZ1, HPD, ALDH8A1, PON1, CYP4F2, TDO2, FTCD, HAO2, SDS, PCK1, TAT, KMO* |
|  | GO:0046395 | carboxylic acid catabolic process | 6.390E-9 | 9.945E-7 | *GSTZ1, HPD, ALDH8A1, PON1, CYP4F2, TDO2, FTCD, HAO2, SDS, PCK1, TAT, KMO* |
|  | GO:0044255 | cellular lipid metabolic process | 6.735E-9 | 9.945E-7 | *ADH6, SOCS2, GBA3, ACSL4, CYP2C8, CYP2C9, ACSM3, RDH16, PEMT, CYP2E1, CYP3A4, CETP, APOF, ALDH8A1, PON1, RDH5, CYP4F2, LCAT, CPS1, HAO2, PCK1, SLC27A5* |
|  | GO:0009074 | aromatic amino acid family catabolic process | 7.776E-9 | 1.088E-6 | *GSTZ1, HPD, TDO2, TAT, KMO* |
|  | GO:0042180 | cellular ketone metabolic process | 9.811E-9 | 1.304E-6 | *ACSL4, RDH16, EGR1, CYP3A4, SRD5A2, NQO1, AKR7A3, ALDH8A1, CYP4F2, TDO2, KMO, BHMT* |
| CC: Cellular Component | | | | | |
|  | GO:0034364 | high-density lipoprotein particle | 5.863E-7 | 1.337E-4 | *SAA4, CETP, APOF, PON1, LCAT* |
|  | GO:1990777 | lipoprotein particle | 1.840E-6 | 1.398E-4 | *SAA4, CETP, APOF, PON1, LCAT* |
|  | GO:0034358 | plasma lipoprotein particle | 1.840E-6 | 1.398E-4 | *SAA4, CETP, APOF, PON1, LCAT* |
|  | GO:0032994 | protein-lipid complex | 2.551E-6 | 1.454E-4 | *SAA4, CETP, APOF, PON1, LCAT* |
|  | GO:0005579 | membrane attack complex | 5.001E-6 | 2.280E-4 | *C6, C7, C9* |
|  | GO:0072562 | blood microparticle | 5.041E-5 | 1.915E-3 | *FCN2, AFM, PON1, C9, PZP, FCN3* |
|  | GO:0005581 | collagen trimer | 8.056E-5 | 2.624E-3 | *FCN2, MBL2, DCN, FCN3, MARCO* |
|  | GO:0005796 | Golgi lumen | 1.461E-4 | 3.432E-3 | *PROZ, GPC3, MUC13, DCN, F9* |
|  | GO:1905286 | serine-type peptidase complex | 1.505E-4 | 3.432E-3 | *FCN2, MBL2, FCN3* |
|  | GO:1905370 | serine-type endopeptidase complex | 1.505E-4 | 3.432E-3 | *FCN2, MBL2, FCN3* |
|  | GO:0031012 | extracellular matrix | 2.140E-4 | 4.123E-3 | *IGFALS, GPC3, SPP2, FCN2, DCN, PZP, FCN3, ECM1, MARCO, F9, CXCL12* |
|  | GO:0030312 | external encapsulating structure | 2.196E-4 | 4.123E-3 | *IGFALS, GPC3, SPP2, FCN2, DCN, PZP, FCN3, ECM1, MARCO, F9, CXCL12* |
|  | GO:0005789 | endoplasmic reticulum membrane | 2.404E-4 | 4.123E-3 | *ACSL4, CYP2C8, CYP2C9, RDH16, PEMT, CYP2E1, CYP3A4, HPD, SQLE, SRD5A2, PON1, RDH5, CYP4F2, FTCD, HSD11B1, SLC27A5* |
|  | GO:0098827 | endoplasmic reticulum subcompartment | 2.532E-4 | 4.123E-3 | *ACSL4, CYP2C8, CYP2C9, RDH16, PEMT, CYP2E1, CYP3A4, HPD, SQLE, SRD5A2, PON1, RDH5, CYP4F2, FTCD, HSD11B1, SLC27A5* |
|  | GO:0042175 | nuclear outer membrane-endoplasmic reticulum membrane network | 3.237E-4 | 4.920E-3 | *ACSL4, CYP2C8, CYP2C9, RDH16, PEMT, CYP2E1, CYP3A4, HPD, SQLE, SRD5A2, PON1, RDH5, CYP4F2, FTCD, HSD11B1, SLC27A5* |
|  | GO:0046930 | pore complex | 5.347E-4 | 7.620E-3 | *C6, C7, C9* |
|  | GO:0062023 | collagen-containing extracellular matrix | 7.041E-4 | 9.444E-3 | *GPC3, SPP2, FCN2, DCN, PZP, FCN3, ECM1, F9, CXCL12* |
|  | GO:0031984 | organelle subcompartment | 1.313E-3 | 1.663E-2 | *ACSL4, CYP2C8, CYP2C9, RDH16, PEMT, CYP2E1, CYP3A4, HPD, SQLE, SRD5A2, AKR7A3, PON1, RDH5, CYP4F2, FTCD, HSD11B1, SLC27A5* |
|  | GO:0031089 | platelet dense granule lumen | 1.793E-3 | 2.151E-2 | *SPP2, ECM1* |
|  | GO:0042827 | platelet dense granule | 4.053E-3 | 4.620E-2 | *SPP2, ECM1* |
| KEGG Pathway | | | | | |
|  | M47618 | tyrosine degradation | 2.718E-6 | 1.640E-4 | *GSTZ1, HPD, TAT* |
|  | M9488 | retinol metabolism | 3.760E-6 | 1.640E-4 | *ADH6, CYP2C8, CYP2C9, RDH16, CYP3A4, RDH5* |
|  | M16894 | complement and coagulation cascades | 5.855E-6 | 1.640E-4 | *MBL2, C6, C7, C9, KLKB1, F9* |
|  | M16794 | metabolism of xenobiotics by cytochrome p450 | 6.370E-6 | 1.640E-4 | *ADH6, GSTZ1, CYP2C8, CYP2C9, CYP2E1, CYP3A4* |
|  | M9257 | drug metabolism cytochrome p450 | 7.512E-6 | 1.640E-4 | *ADH6, GSTZ1, CYP2C8, CYP2C9, CYP2E1, CYP3A4* |
|  | M47880 | regulation of complement cascade mac inhibition | 2.240E-5 | 3.261E-4 | *C6, C7, C9* |
|  | M47878 | lectin pathway of coagulation cascade fibrinogen to fibrin | 3.185E-5 | 4.172E-4 | *FCN2, MBL2, FCN3* |
|  | M2920 | linoleic acid metabolism | 3.653E-5 | 4.350E-4 | *CYP2C8, CYP2C9, CYP2E1, CYP3A4* |
|  | M766 | glycine serine and threonine metabolism | 4.791E-5 | 4.828E-4 | *CTH, GNMT, SDS, BHMT* |
|  | M10911 | cysteine and methionine metabolism | 6.955E-5 | 6.508E-4 | *CTH, SDS, TAT, BHMT* |
|  | M5410 | arachidonic acid metabolism | 5.643E-4 | 4.348E-3 | *CYP2C8, CYP2C9, CYP2E1, CYP4F2* |
|  | M11521 | glycolysis gluconeogenesis | 7.273E-4 | 5.293E-3 | *ADH6, FBP1, ENO3, PCK1* |
|  | M980 | tryptophan metabolism | 2.274E-3 | 1.568E-2 | *OGDHL, TDO2, KMO* |
|  | M47979 | nad biosynthesis | 2.700E-3 | 1.768E-2 | *TDO2, KMO* |
|  | M14933 | steroid hormone biosynthesis | 5.625E-3 | 3.204E-2 | *CYP3A4, SRD5A2, HSD11B1* |
|  | M16817 | oocyte meiosis | 6.514E-3 | 3.413E-2 | *AURKA, PTTG1, CCNB2, CDC20* |
|  | M47582 | spindle assembly checkpoint signaling | 8.267E-3 | 4.165E-2 | *PTTG1, CDC20* |
|  | M7963 | cell cycle | 9.240E-3 | 4.483E-2 | *PTTG1, CCNB2, MCM4, CDC20* |
|  | M4629 | nitrogen metabolism | 9.876E-3 | 4.620E-2 | *CTH, CPS1* |
|  | M13088 | PPAR signaling pathway | 1.052E-2 | 4.752E-2 | *ACSL4, PCK1, SLC27A5* |


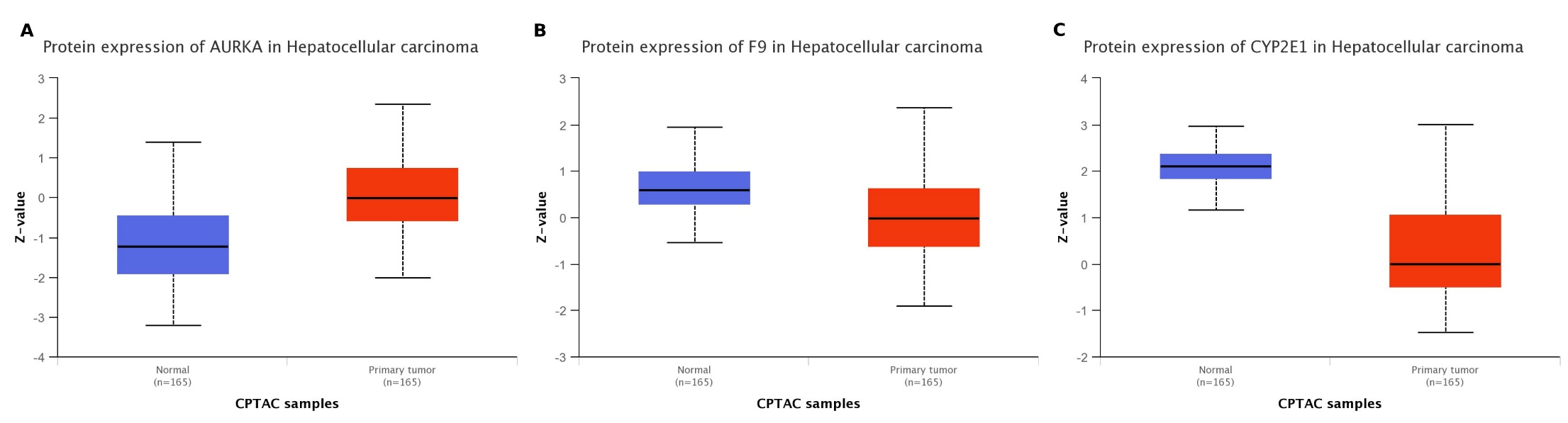


**Figure S1.** Protein expression profiles of hub genes between normal liver tissues and LIHC tissues. The results were obtained using the UALCAN database**.** The protein expression profiles of *AURKA* **(A)**, *F9* **(B)**, and *CYP2E1* **(C)**. Blue bars represents normal tissues, red bars represents tumor tissues. **CPTAC**; Clinical Proteomic Tumor Analysis Consortium, **UALCAN**; University of Alabama Cancer Database (<https://ualcan.path.uab.edu/>, accessed on 12 May 2024), **Z-value**; standard deviations from the median across samples for the given cancer type.
